# Influence of age on real-life effects of doxycycline for acute exacerbations among COPD outpatients: a population-based cohort study

**DOI:** 10.1136/bmjresp-2019-000535

**Published:** 2020-02-18

**Authors:** Yuanyuan Wang, Jens H Bos, H Marike Boezen, Jan-Willem C Alffenaar, J F M van Boven, Catharina C M Schuiling-Veninga, Bob Wilffert, Eelko Hak

**Affiliations:** 1 Department of PharmacoTherapy, Epidemiology & Economics, Groningen Research Institute of Pharmacy, University of Groningen, Groningen, The Netherlands; 2 Department of Epidemiology, University Medical Center Groningen, University of Groningen, Groningen, The Netherlands; 3 Department of General Practice & Elderly Care Medicine, Groningen Research Institute for Asthma and COPD (GRIAC), University Medical Center Groningen, University of Groningen, Groningen, The Netherlands; 4 Department of Clinical Pharmacy & Pharmacology, University Medical Center Groningen, University of Groningen, Groningen, The Netherlands

**Keywords:** respiratory infection, infection control, COPD exacerbations

## Abstract

**Introduction:**

Although bacteria contribute significantly to acute exacerbations of chronic obstructive pulmonary disease (AECOPD), the added value of antibiotics remains controversial, especially in outpatient settings. Age may affect antibiotic effectiveness, but real-world evidence is lacking. We aimed to assess the influence of age on the effectiveness of doxycycline for AECOPD.

**Methods:**

A retrospective cohort study among outpatients with the first recorded AECOPD treated with oral corticosteroids was conducted using a large pharmacy dispensing database. The primary outcome was treatment failure within 15–31 days after treatment start. Secondary outcome was time to second exacerbation. All analyses were stratified by age groups.

**Results:**

We identified 6300 outpatients with the first AECOPD. 2261 (36%) received doxycycline and 4039 (64%) did not receive any antibiotic (reference group). Overall, there was no difference in treatment failure (adjusted OR: 0.97, 95% CI: 0.84 to 1.12) between two groups. Similarly, no difference in treatment failure was observed in younger groups. However, in patients with advanced age (≥75 years), treatment failure was significantly reduced by doxycycline compared with reference (16% vs 20%, adjusted OR: 0.77, 95% CI: 0.62 to 0.97). Overall, median time to second exacerbation was 169 days (95% CI: 158 to 182 days) in doxycycline group compared with 180 days (95% CI: 169 to 191 days) in reference group (adjusted HR: 1.06, 95% CI: 0.99 to 1.12). Although in older patients there was a trend within 3 months towards longer time of next exacerbation by doxycycline, it did not achieve statistical significance.

**Conclusions:**

Our findings showed short-term treatment benefit of doxycycline added to oral corticosteroids for chronic obstructive pulmonary disease patients with advanced age. This value remains unclear for persons aged under 75 years in current primary care. Long-term preventive benefits of doxycycline for the next exacerbation were not observed, irrespective of age.

**Key messages**

- What are the real-world effects of doxycycline treatment on acute exacerbations of chronic obstructive pulmonary disease (AECOPD) in an outpatient setting and does age make any difference?
- There are significant benefits of doxycycline in reducing treatment failure of AECOPD among older outpatients. This benefits were not found in those aged under 75 years. Long-term preventive benefit of doxycycline for next exacerbation was not observed, irrespective of age.
- This large cohort study evaluated doxycycline effects on AECOPD in both short-term and long-term for outpatients based on real-world data and highlights the possible influence of age on short-term effects of doxycycline.

## Introduction

Chronic obstructive pulmonary disease (COPD) is a chronic, progressive, inflammatory disease and a leading cause of death worldwide.^1^ Acute exacerbations of COPD (AECOPD) characterised by the sudden worsening of respiratory symptoms may accelerate the progress of COPD and contribute significantly to worsened patients’ health status, mortality and medical costs.^2 3^ As about 50% of AECOPD are triggered by bacterial infections,^4^ the use of antibiotics has become a common component in the therapeutic management of AECOPD.^5 6^

The evidence on the benefits of oral corticosteroids for AECOPD is of high quality.^6 7^ However, the effects of antibiotics in addition to corticosteroids are still uncertain, especially in an outpatient setting. A Cochrane review in 2012 did not show a significant reduced risk of treatment failure by antibiotics.^8^ Although treatment guidelines in 2017 conditionally recommended antibiotics for AECOPD among outpatients,^6^ this recommendation was based on synthesised evidence from only two earlier randomised controlled trials (RCTs).^9 10^ In the same year, a new large RCT concluded that antibiotics for AECOPD in an outpatient setting are not effective.^11^ Later in 2018, an updated Cochrane review included two more RCTs than in 2012^11 12^ and showed statistically significant beneficial effects of antibiotics.^13^ Of note, while most RCTs focused on the short-term effect of antibiotics, the long-term effect in outpatient settings also remains unclear due to conflicting results.^11 14 15^

The majority of AECOPD is treated in primary care and establishing a bacterial infection diagnosis with sputum cultures is not always feasible in routine practice due to technical reasons.^5 16^ Therefore, accurate prescribing of antibiotics according to guidelines is still low.^17–19^ Notably, many studies indicate that the susceptibility to infections increases with age.^20 21^ According to a large population-based observational study, the protective effect of antibiotics against pneumonia is more pronounced in older patients.^22 23^ Thus, we hypothesised that older patients may benefit more from empirical antibiotic treatment for AECOPD than younger patients.

In addition to prednisone or prednisolone, doxycycline is one of the first-choice oral antibiotics for AECOPD if antibiotic treatment is indicated.^5 16 24^ Since only one RCT studied doxycycline, we conducted a cohort study to evaluate if doxycycline has meaningful value added to oral corticosteroids on AECOPD in both the short-term and longer-term for outpatients, and examined the potential effect modification across age groups.

## Methods

### Study design and data source

**Figure 1:**
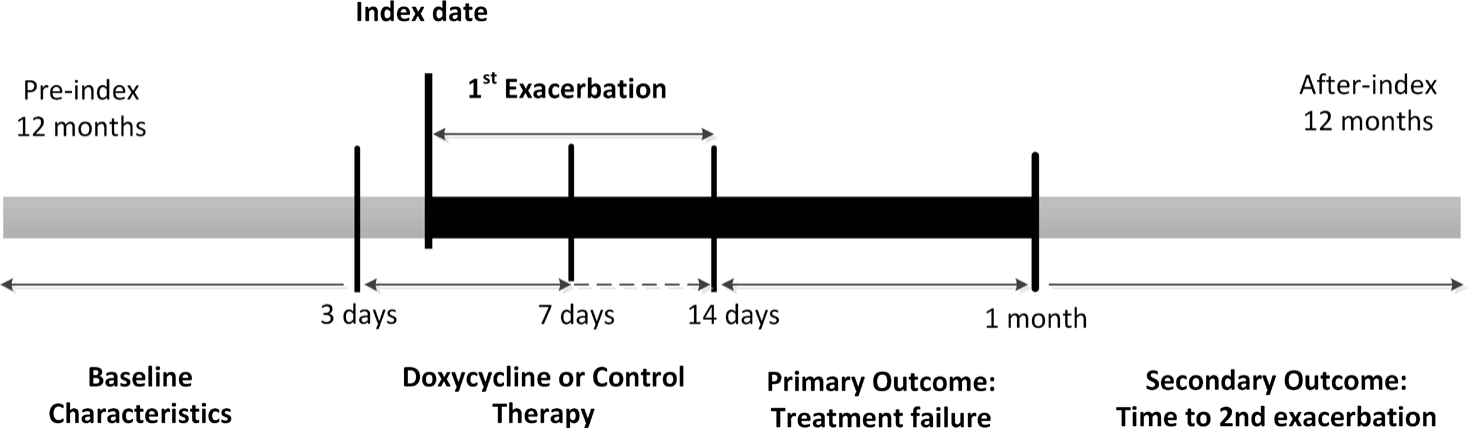
Retrospective cohort study design.

We applied a retrospective inception cohort study (figure 1) using the University of Groningen’s prescription database IADB.nl that contains over 1.2 million dispensings from about 600 000 patients in 60 community pharmacies in the Netherlands since 1994.^25 26^ IADB.nl provided complete information including date of birth, gender, prescribed drug name, anatomical therapeutic chemical (ATC) codes, dispensing date, quantity dispensed and dose regimen.^27^ Over-the-counter (OTC) drugs and drugs dispensed during hospitalisation are not available in the database. As Dutch patients practically always register at a single community pharmacy, the patient’s drug prescription history is usually complete.^28^ Data from January 1994 to December 2015 were used for this study, which was conducted and reported according to checklists of Strengthening the Reporting of Observational Studies in Epidemiology guidelines (online supplementary material).

10.1136/bmjresp-2019-000535.supp1 Supplementary data

### Study population

COPD outpatients with first recorded AECOPD were included in this study. We selected eligible patients according to the following inclusion criteria: (1) presence of COPD identified based on at least two COPD-related drug prescriptions (online supplementary table S1) within 1 year before index date.^5 24^ The date of first recorded AECOPD during the study period was set as index date. (2) The experience of the first recorded AECOPD, which was defined by the prescription of high dose prednisone (ATC-code H02AB07) or prednisolone (H02AB06) short courses (a daily dose of 40 mg for 5 days or a daily dose of 30 mg for 7 days with maximum extension of 14 days) according to treatment guidelines.^14 24 29^ (3) Registration in the IADB.nl for at least 2 years before and 1 year after the index date. (4) Receipt of doxycycline or either received any antibiotics 3 days before till 7 days after the index date. Furthermore, we excluded patients who met the following exclusion criteria: (1) Receipt of another antibiotic treatment than doxycycline 3 days before till 7 days after the index date. (2) Age under 55 years, to reduce the chance of including possible asthma patients.^30 31^ Age was calculated using the difference between index date and birth date. (3) Presence of potential immunocompromised diseases, which were defined by the prescription of antiviral drugs for HIV infection, immunosuppressant drugs or antineoplastic agents within 1 year before index date and 1 month after index date.

### Exposure and outcomes

Among patients with a first identified AECOPD, during their treatment period of oral prednisone or prednisolone (3 days before till 7 days after the index date), those who were also prescribed doxycycline and no other antibiotics were classified as treatment exposure group. Those who did not receive doxycycline (or any other antibiotic) were classified as reference group. The primary outcome was treatment failure defined as a new prescription of prednisone or prednisolone or an antibiotic treatment within a period of 15–31 days after index date according to Dutch NHG (Nederlands Huisartsen Genootschap) guidelines for COPD management. Secondary outcome was time to the second exacerbation within a follow-up period of 12 months. As the first exacerbation may last for a longer time, and to avoid counting it as second exacerbation, we limited the minimum time from first exacerbation to second one to 21 days.^32^ A few patients could be included in the treatment failure outcome and the second exacerbation outcome if the drugs appeared within 21 and 31 days after index date.

### Covariates

The following covariates were included as potential confounders: age; gender; frequently used maintenance drugs for COPD treatment within 365 days before index date including SABA, SAMA (short-acting muscarinic antagonist), LABA (ong-acting β agonist), LAMA (long-acting muscarinic antagonist), SABA/SAMA, LABA/LAMA, LABA/ICS (inhaled corticosteroid) and theophylline. Comorbidities in COPD patients were defined on the basis of at least two prescriptions of related drugs within 365 days prior to index date: diabetes (A10), heart failure (C01AA05 or C03C), ischaemic heart disease (C01DA), other cardiovascular disease (C02 or C03 or C07 or C08 or C09, but not for C01AA05, C03C and C01DA), dyslipidaemia (C10), osteoporosis (M05B), anxiety (N05B, N05C), dementia (N06D), depression (N06A), rheumatic arthritis (M01 or M02) and hypothyroid disease (H03).^30^

### Statistical methods

The differences in distribution of baseline characteristics of COPD outpatients between two exposure groups were compared using t-test and χ^2^ test for continuous and categorical variables, respectively. We applied logistic regression to estimate the OR with 95% CI for treatment failure and adjusted for possible covariates. The time to second exacerbation was compared by Kaplan-Meier survival analysis. Cox proportional hazards regression was applied to estimate the HR and 95% CI for risk of second exacerbation. For all tests, p-values were two-sided. A p-value<0.05 was considered statistically significant. All analyses were performed using IBM SPSS statistics V.22.

### Sensitivity analysis

To further assess the robustness of our results, we performed several sensitivity analyses. Treatment failure was defined by the use of prednisone, prednisolone or antibiotics according to Dutch guidelines.^24^ However, considering that not all antibiotics are used for acute exacerbations, we narrowed the outcome definition by including only frequently prescribed antibiotics among COPD patients in Netherlands (online supplementary table S2) based on frequencies in the IADB database and previous published paper.^14 27^ In addition, we further narrowed the definition of treatment failure by including prednisone or prednisolone only to see if there is any difference with definition by including antibiotics only. Third, considering the COPD treatment may change during the long period of study time, we did a sensitivity analysis by limiting our study period to the last 10 years and compared the result with those from previous decade.

### Patient and public involvement

Patients were involved in the study indirectly, due to the nature of the study, written consent was not required. IADB.nl data are collected in accordance with the national and European guidelines on privacy requirements for handling human data.

## Results

### Study participants

In total, 8889 COPD patients with a first recorded AECOPD were identified, all received prednisone or prednisolone. Of those, we excluded 2589 patients who were prescribed another antibiotic than doxycycline, that is, our exposure of interest. Of the remaining 6300 patients, 2261 patients who received doxycycline were included as treatment group, and the remaining 4039 patients who did not receive any antibiotic were included as reference group (figure 2). The baseline characteristics of both comparison groups are summarised in table 1. The two groups were balanced for most characteristics. However, the mean age in the doxycycline group was slightly higher than the reference group. A little higher prevalence of LABA/ICS and doxycycline prescriptions and lower prevalence of prescriptions of SABA were seen in the doxycycline group compared with reference.

**Figure 2:** Flow chart of participation selection. COPD, chronic obstructive pulmonary disease.

**Table 1:** Baseline characteristics of COPD outpatients with first exacerbation in treatment groups.

	Doxycycline (n=2261)	Reference (n=4039)	P value
Gender, no. (%)			
Male	1085 (48.0)	1999 (49.5)	0.252
Female	1176 (52.0)	2040 (50.5)	
Age, years, no. (%)			
Mean age (SD)	71.08 (9.6)	70.30 (9.4)	0.002*
55–64	667 (29.5)	1285 (31.8)	0.018†
65–74	733 (32.4)	1357 (33.6)	
≥75	861 (38.1)	1397 (34.6)	
Year of index date (%)			
1996–2004	893 (39.5)	1676 (41.5)	0.121
2005–2015	1368 (60.5)	2363 (58.5)	
Prescriber			
GP	2147 (95.0)	3424 (84.8)	<0.001
Specialist	114 (5.0)	615 (15.2)	
Maintenance medicines, no. (%)			
SABA	775 (34.3)	1579 (39.1)	<0.001
LABA	494 (21.8)	847 (21.0)	0.414
SAMA	689 (30.5)	1216 (30.1)	0.761
LAMA	555 (24.5)	1020 (25.3)	0.534
SABA/SAMA	80 (3.5)	173 (4.3)	0.149
LABA/LAMA	0 (0)	1 (0)	0.454
LABA/ICS	1093 (48.3)	1846 (45.7)	0.044
Theophylline	124 (5.5)	159 (3.9)	0.004
Comorbidity, no. (%)			
Diabetes mellitus	301 (13.3)	504 (12.5)	0.341
Disorders of lipid metabolism	629 (27.8)	1093 (27.1)	0.517
Heart failure	363 (16.1)	676 (16.7)	0.484
Ischaemic heart disease	206 (9.1)	336 (8.3)	0.282
Other cardiovascular disorders	843 (37.3)	1493 (37.0)	0.801
Thyroid disease	115 (5.1)	192 (4.8)	0.556
Rheumatic arthritis	355 (15.7)	660 (16.3)	0.508
Osteoporosis	117 (5.2)	232 (5.7)	0.343
Anxiety	392 (17.3)	649 (16.1)	0.193
Depression	274 (12.1)	438 (10.8)	0.125
Dementias	9 (0.4)	10 (0.2)	0.296

*Student’s t-test.

†Pearson χ^2^ test;.

COPD, chronic obstructive pulmonary disease; GP, general practitioner; ICS, inhaled corticosteroid; LABA, long-acting β agonist; LABA/ICS, long-acting β agonist/inhaled corticosteroid; LABA/LAMA, long-acting β agonist/long-acting muscarinic antagonist combinations; LAMA, long-acting muscarinic antagonist; SABA, short-acting β agonist; SABA/SAMA, short-acting β agonist/short-acting muscarinic antagonist combinations; SAMA, short-acting muscarinic antagonist.

### Primary outcome

Between 15 and 31 days after the first exacerbation, 354 (15.7 %) patients in the doxycycline group versus 640 (15.8 %) patients in the reference group had treatment failure (crude OR: 0.99 (95% CI: 0.89 to 1.14), table 2). After adjustment for potential confounders, there still was no statistical difference between the two groups regarding the rate of treatment failure, the adjusted OR (aOR) of treatment failure was 0.97 (95% CI: 0.84 to 1.12).

**Table 2:** OR for treatment failure of first exacerbation among COPD outpatients in different age groups

	Doxycycline (n=2261)	Reference (n=4039)	Crude OR (95% CI)	Adjusted OR* (95% CI)
Treatment failure (n, %)				
Overall	354 (15.7)	640 (15.8)	0.99 (0.86 to 1.14)	0.97 (0.84 to 1.12)
Subgroups				
55–65	99 (14.8)	166 (12.9)	1.18 (0.90 to 1.54)	1.17 (0.89 to 1.53)
65–75	116 (15.8)	196 (14.4)	1.11 (0.87 to 1.43)	1.11 (0.86 to 1.42)
≥75	139 (16.1)	278 (19.9)	0.78 (0.62 to 0.97)	0.77 (0.62 to 0.97)

*Adjusted for age, SABA, LABA/ICS, theophylline.

COPD, chronic obstructive pulmonary disease.

In the analysis stratified by age groups, there was no significant difference in the rate of treatment failure between the two treatments for age groups below 75 years old. However, for COPD outpatients aged 75 years and older, less patients in the doxycycline group experienced treatment failure than in the reference group (16.1% vs 19.9%, OR: 0.78 (95% CI: 0.62 to 0.97)). After adjustments for possible confounders, the value of OR did not change much, and results were compatible with a 23% relative risk reduction of treatment failure observed by doxycycline treatment compared with reference group (aOR: 0.77 (95% CI: 0.62 to 0.97)).

### Secondary outcome

**Figure 3:**
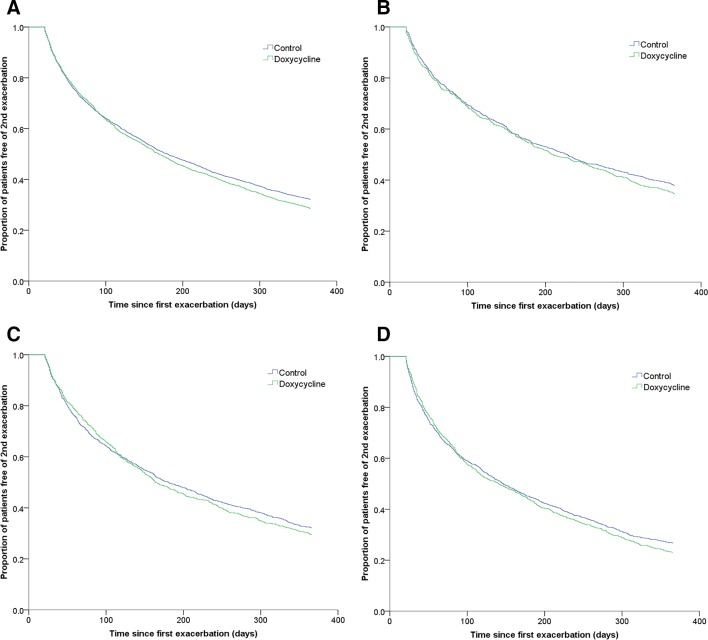
Kaplan-Meier curves showing the proportion of patients free of second exacerbation in COPD outpatients up to 12 months’ follow-up: (A) all-age group patients (p=0.07); (B) patients aged 55–64 (p=0.252); (C) patients aged 65–74 (p=0.564); (D) patients aged ≥75 (p=0.421). COPD, chronic obstructive pulmonary disease.

After a follow-up of 12 months, 71.4% and 67.9% COPD outpatients experienced the next exacerbation in doxycycline and reference groups, respectively. The median time to next exacerbation was 169 days (95% CI: 156 to 182) in the doxycycline group compared with 180 days (95% CI: 169 to 191) in the reference group (p=0.07, figure 3). However, if we included only those patients who experienced a second exacerbation within 12 months follow-up, the median time was longer in the doxycycline group than in the reference group, though it was not statistically significant (97 days (95% CI: 91 to 103) versus 91 days (95% CI: 86 to 96), p=0.128).

When the results were stratified according to different age groups, we did not find significant differences, although older people (aged 65–74 and ≥75) on doxycycline experienced a lower risk of next exacerbation than the reference group at early time points (within 3 months) of the follow-up (figure 3). However, we found that in both treatment groups, the median time to second exacerbation was shorter (p<0.01) in older age groups compared with younger age groups (online supplementary table S3 and figure S1).

Overall, around 30%, 50% and 70% patients in both treatment groups experienced a new exacerbation in the 3, 6 and 12 months follow-up, respectively (online supplementary table S4). From the univariate Cox regression model, the risk for the next exacerbation was similar between two treatment groups, the HR (doxycycline vs reference) was 1.00 (95% CI: 0.9 to 1.09), 1.03 (95% CI: 0.96 to 1.11) and 1.07 (95% CI: 1.00 to 1.14) in 3, 6 and 12 months follow-up. Similar results were observed after adjusting for potential confounding factors, the HR was 0.98 (95% CI: 0.89 to 1.07), 1.02 (95% CI: 0.95 to 1.09) and 1.06 (95% CI: 0.99 to 1.12), respectively.

### Sensitivity analysis

When we further defined the primary outcome of treatment failure including only frequently used antibiotics, it showed consistent results in that doxycycline did not reduce treatment failure for the overall cohort (aOR: 0.99 (0.85 to 1.14)), but that doxycycline treatment showed benefits in patients 75 years or older with 137 patients (15.9 %) and 268 patients (19.2 %) that experienced treatment failure in the doxycycline group and the reference group, respectively (aOR: 0.80 (0.63 to 1.00)) (online supplementary table S5). When we further narrow our treatment failure definition to a new prescription of prednisone or prednisolone, we also observed reduced treatment failure by doxycycline in the older age group compared with reference (aOR: 0.72 (0.55 to 0.95), online supplementary table S6), while no significant difference was observed between groups for the narrow definition of treatment failure by a new prescription of antibiotics. When we limit the study period to the last decade (2005–2015) and the previous decade (1994–2004) separately, the treatment failure was also less among patients with advanced age in doxycycline group than reference group (aOR: 0.75 (0.55 to 1.01) and aOR: 0.84 (0.60 to 1.18), separately, online supplementary table S7).

## Discussion

### Main findings

In a real-world population of primary care patients with AECOPD of any age, doxycycline did not appreciably reduce the failure rate, nor prolong time to next exacerbation. However, when stratified by age, we found a statistically significant 23% relative reduction in treatment failure by doxycycline for AECOPD in outpatients aged 75 years and older. These benefits were not seen in younger age groups. In the long-term, we observed that the protective effect of doxycycline for the subsequent exacerbations was only present in the first 3 months among older patients. After that, the protective effect wanes over time.

The observed short-term effect regarding reduction rate in treatment failure for older patients (≥75 years) is compatible with a previous RCT which found that short-term treatment non-response rates are significantly lower in the doxycycline group compared with placebo (OR: 0.77, 95% CI (0.63 to 0.94)).^11^ Our subgroup result is also consistent with a recent Cochrane review that showed that the current available antibiotics reduce the risk for treatment failure between 7 days and 1 month after treatment initiation (OR: 0.72, 95% CI (0.56 to 0.94)).^13^

The benefit of doxycycline in older patients may be primarily due to their increased susceptibility to infection.^20^ With increasing age, not only the lung function changes, the natural defence mechanisms of the lungs also decrease gradually.^33^ Intercellular communications become less effective which could contribute to immune-senescence.^34^ Additionally, mucocilliary clearance is also compromised with age.^35^ All these changes with age contribute to the greater possibility of bacterial infection and inflammation in elderly.^20^ Therefore, the elderly seem to benefit more from antibiotic treatment than younger patients.

The average age of study patients (about 70 years) was comparable with previous studies.^36^ We did not find a short-term benefit of doxycycline in the younger age group (<75), which may be explained by the fact that the overall rate of appropriate antibiotic use in practice is rather low.^17–19^ According to GOLD (Global Initiative for Chronic Obstructive Lung Disease), general practitioners should only consider antibiotics for patients when signs of bacterial infection are present.^5^ However, in reality, guidelines regarding the prescription of antibiotics are poorly followed,^17 19^ on average in only 25% of AECOPD antibiotics were prescribed properly according to the GOLD criteria.^18^ For AECOPD with other aetiology like viral infection and environmental pollution, antibiotics may not have been effective. Of note, a complicating factor in the outpatient setting is that sputum cultures are not feasible as they take at least 2 days and frequently do not give reliable results.^5^ Identification of bacterial exacerbation still relies on clinical assessment rather than laboratory biomarkers.^37^ As infection is the most treatable cause of breathlessness, it is not surprising that many patients continue to receive antimicrobials in the absence of clinical, pathological or radiological evidence of infection.^38^ Therefore, if the proportion of patients who were prescribed doxycycline but in fact should not be given antibiotics is large, it will be difficult to find significant beneficial effects of doxycycline treatment.

Observed long-term effects from this study for all patients independent of age were also consistent with the findings of two previous RCTs that antibiotics did not prolong time to next exacerbation.^11 39^ However, two observational studies showed different results in that the time to next exacerbation was significantly extended if the exacerbation was treated with antibiotics.^14 15^ Similarly, one RCT also showed a prolonged time to next exacerbation by antibiotic treatment.^9^ In this study, the prolonged time to next exacerbation by doxycycline was only seen in older outpatients within 3 months. Of note, as different definitions for subsequent exacerbation and different types of antibiotics were used in these studies when evaluating the long-term effect of antibiotics, these may lead to the inconsistent results.

Besides the effects of ageing on bacterial susceptibility,^22^ we should also realise that COPD itself is an age-related chronic inflammatory disorder. After the lungs reach their maximum function around the age of 25 years, its function progressively declines as a sequence of structural and physiological changes to the lung.^33^ With ageing, severity and comorbidities of COPD usually also increase. These factors could further influence the frequency of exacerbations in primary care patients with COPD.^40^ A higher frequency also means a shorter time to experience the next exacerbation. In this study, we have found that the time to next exacerbation was shorter in older than younger patients, and it was consistent in both doxycycline and reference groups.

### Strengths and limitations

This study has several strengths. One strength is that this study was based on a large real-life prescription database which enabled us to evaluate the effects of doxycycline in a large COPD population. Another strength is that both short-term and long-term effects of additional doxycycline were evaluated, which may offer more comprehensive support for decision-making in clinical practice. Additionally, we chose the first recorded exacerbation as investigated event for all COPD outpatients, which could exclude the influence of historical exacerbation frequency as a risk factor on targeted outcomes to a large extent. In addition, as oral steroids and antibiotics cannot be bought OTC in the Netherlands, the study population from the IADB database represents a generalisable population for AECOPD outpatients treated with doxycycline.

Limitations to observational studies also need to be discussed. First, due to the characteristics of the prescription database, there was no diagnostic information available. Therefore, the definition of COPD, comorbidities and outcomes were defined using related drugs as proxies, which may result in potential misclassification bias. Second, although the relevant measured baseline characteristics of the two groups were similar in this study, other clinical information like lung function, GOLD stages (I–IV) of COPD and severity of exacerbations were lacking, which may influence our outcome to some extent if these unknown characteristics were not balanced between the two study groups. In clinical practice, antibiotics may be prescribed to those who in fact did not have enough indication of infection due to limitation of outpatients setting or to those with more severe COPD,^5^ which may have led to underestimation of the efficacy of additional doxycycline treatment in all age groups compared with corticosteroids only. Third, there were overlap for a few patients within 21 days and 31 days between the short-term and long-term outcome definitions by a new prescription of corticosteroids due to lack of clinical information to distinguish and classify the outcomes. Finally, although we set the age limitation of 55 years older to exclude potential asthma, asthma-COPD overlap patients may still existed as we did not exclude the patients who use asthma drugs at the stage of study design. However, these patients were very few and unlikely to influence the overall results based on the fact that no patients were prescribed leukotriene receptor antagonists and only 11 patients were prescribed cromoglycates within 1 year before index date among all the AECOPD patients in our study.

### Implications for future research and clinical practice

The tendency towards better effects of antibiotics in the elderly COPD patients may offer clues for clinicians and researchers for more targeted management of AECOPD. In particular, decision-making about empirical antibiotic therapy for AECOPD should take the age of patients into consideration. However, before that, more prospective, well-designed studies with more accurate diagnostic information are needed to further confirm the finding from this study.

Although related guideline and GOLD report about antibiotic use for AECOPD were basically based on secondary care RCT evidence,^5 6^ decision-making in daily practice is influenced by many factors making AECOPD treatment more challenging in outpatient settings.^5^ Therefore, identifying high risk populations for infection may improve management and clinical decisions about antibiotic use in COPD outpatients. The high risk of infection and beneficial effects from antibiotics for AECOPD in elderly outpatients should warrant a personalised approach towards antibiotic treatment.

## Conclusion

Doxycycline in addition to oral corticosteroid treatment was associated with a reduced risk of treatment failure for AECOPD in patients 75 years or older, but not in younger patients. Long-term effects of doxycycline treatment on subsequent exacerbations was not observed, though among older persons there was a non-statistically significant beneficial trend within 3 months after doxycycline treatment. Clinicians should take the age of patients into consideration in empirical antibiotic therapy for AECOPD. More real-world studies with high quality, preferably prospective clinical data collections, should be recommended to confirm the influence of age on effects of antibiotics and to further explore which patient groups could benefit most from antibiotic treatment for AECOPD.
