# Supplementary material for: Influence of age on real-life effects of doxycycline for acute exacerbations among COPD outpatients: a population-based cohort study
Source: BMJ Open Respir Res. 2020 Feb 18;7(1):e000535. doi: 10.1136/bmjresp-2019-000535 (PMC7047485; doi:10.1136/bmjresp-2019-000535)
Supplement: Supplementary data [file bmjresp-2019-000535supp001.pdf]

Supplementary Material

Supplementary Tables

Table S1. Maintenance Drugs that prescribed for COPD according to guidelines.

No.	Maintenance drugs	Subgroups	ATC codes
1	SABA	salbutamol	R03AC02
		terbutaline	R03AC03
2	SAMA	ipratropium	R03BB01
3	LABA	formoterol	R03AC13
		salmeterol	R03AC12
		indacaterol	R03AC18
		olodaterol	R03AC19
4	LAMA	tiotropium	R03BB04
		aclidinium	R03BB05
		glycopyrronium	R03BB06
		umeclidinium	R03BB07
5	Combination of SABA plus SAMA	fenoterol/ipratropium	R03AL01
		salbutamol/ipratropium	R03AL02
6	Combination of LABA plus LAMA	vilanterol/umeclidinium	R03AL03
		indacterol/glycopyrronium	R03AL04
		formoterol/aclidinium	R03AL05
		olodateral/tiotropium	R03AL06
7	Combination of LABA plus ICS	salmeterol/fluticasone	R03AK06
		formoteral/budesonide	R03AK07
		formoterol/beclomethasone	R03AK08
		vilanterol/fluticasone furoate	R03AK10
		formoterol/fluticasone	R03AK11
8	Theophylline		R03DA04

SABA: short-acting beta-2-agonist; SAMA: short-acting muscarinic antagonist; LABA: long-acting beta-2-agonist; LAMA: long-acting muscarinic antagonist; ICS: Inhaled Coticosteroids;

Table S2. Antibiotics that are frequently prescribed among COPD patients. *

No.	Antibiotics	ATC codes
1	Doxycycline	J01AA02
2	Amoxicillin and enzyme inhibitor (co-amoxiclav)	J01CR02
3	Amoxicillin	J01CA04
4	Clarithromycin	J01FA09
5	Azithromycin	J01FA10
6	Ciprofloxacin	J01MA02
7	Sulfamethoxazole and trimethoprim (co-trimoxazole/ TMP/SMX)	J01EE01
8	Moxifloxacin	J01MA14
9	Nitrofurantoin	J01XE01
10	Levofloxacin	J01MA12
11	Roxithromycin	J01FA06
12	Trimethoprim	J01EA01
13	Ofloxacin	J01MA01
14	Erythromycin	J01FA01

*Which was based on two Cochrane reviews[4] and use within the University Groningen prescription database IADB.nl from Netherlands (http://www.iadb.nl/).

Table S3. The median time to second exacerbation in doxycycline and reference groups stratified by age groups.

	**Doxycycline (days, 95% CI)**	**Reference (days, 95% CI)**
**Overall**	169 [156, 182]	180 [169, 191]
**55-64**	210 [178, 242]	228 [203, 253]
**65-74**	164 [144, 184]	181 [161, 201]
**≥75**	141 [121, 161]	147 [131, 163]

CI: confidence interval.

Table S4. Cox proportional hazards analysis for the second exacerbation in COPD outpatients stratified by age groups and follow-up times.

	**Doxycycline (N=2261; n/N, %)**	**Reference (N=4039; n/N, %)**	**Crude HR (95% CI)**	**Adjusted HR* (95% CI)**
**Up to 3 months' follow up**				
Overall	764 (33.8)	1362 (33.7)	1.00 [0.91, 1.09]	0.98 [0.89, 1.07]
Age groups				
55-64	190 (28.5)	360 (28.0)	1.03 [0.87, 1.23]	1.01 [0.84, 1.20]
65-74	232 (31.7)	456 (33.6)	0.92 [0.79, 1.08]	0.92 [0.79, 1.08]
≥75	342 (39.7)	546 (39.1)	1.00 [0.87, 1.14]	1.00 [0.87, 1.14]
**Up to 6 months' follow up**				
Overall	1167 (51.6)	2020 (50.0)	1.03 [0.96, 1.11]	1.02 [0.95, 1.09]
Age groups				
55-64	303 (45.4)	574 (44.7)	1.03 [0.90, 1.19]	1.01 [0.88, 1.16]
65-74	381 (52.0)	677 (49.9)	1.03 [0.91, 1.17]	1.03 [0.91, 1.17]
≥75	483 (56.1)	769 (55.0)	1.01 [0.90, 1.13]	1.01 [0.90, 1.13]
**Up to 12 months' follow up**				
Overall	1614 (71.4)	2741 (67.9)	1.07 [1.00, 1.14]	1.06 [0.99, 1.12]
Age groups				
55-64	436 (65.4)	798 (62.1)	1.08 [0.96, 1.21]	1.05 [0.94, 1.18]
65-74	516 (70.4)	920 (67.8)	1.05 [0.94, 1.17]	1.05 [0.94, 1.17]
≥75	662 (76.9)	1023 (73.2)	1.06 [0.96, 1.17]	1.06 [0.96, 1.17]

*Adjusted for age, SABA, LABA/ICS and theophylline. HR: hazards ratio.

Table S5. Sensitivity analysis: Odds ratio for treatment failure of first exacerbation in COPD outpatients stratified by age groups.

	Doxycycline (n=2261)	Reference (n=4039)	Crude OR (95% CI)	Adjusted OR* (95% CI)	P-value
**Treatment failure# (n, %)**					
**Overall**	346 (15.3)	618 (15.3)	1.00 [0.87, 1.15]	0.99 [0.85, 1.14]	0.84
**Subgroups**					
55-64 (1952)	96 (14.4)	161 (12.5)	1.17 [0.89, 1.54]	1.17 [0.89, 1.54]	0.26
65-74 (2090)	113 (15.4)	189 (13.9)	1.13 [0.88, 1.45]	1.12 [0.87, 1.45]	0.38
≥75 (2258)	137 (15.9)	268 (19.2)	0.80 [0.64, 1.00]	0.80 [0.63, 1.00]	0.05

#Which was defined by a narrow way that patients were given a new prescription of prednisone or prednisolone or a frequently used antibiotic that listed in Table S1 within a period of 15 to 31 days after index date.

OR = odds ratio; CI = confidence interval; *Adjusted for age, SABA, LABA/ICS and theophylline.

Table S6. Sensitivity analysis: odds ratio for treatment failure of first exacerbation among COPD outpatients in different age groups.

	Doxycycline (n=2261)	Reference (n=4039)	Crude OR (95% CI)	Adjusted OR* (95% CI)
**Treatment failure (n, %)** #				
**Overall**	221 (9.8)	399 (9.9)	0.99 [0.83, 1.18]	0.97 [0.81, 1.15]
**Subgroups**				
**55-65**	61 (9.1)	99 (7.7)	1.21 [0.86, 1.68]	1.20 [0.86, 1.67]
**65-75**	74 (10.1)	116 (8.5)	1.20 [0.88, 1.63]	1.20 [0.88, 1.63]
**≥75**	86 (10.0)	184 (13.2)	0.73 [0.56, 0.96]	0.72 [0.55, 0.95]
**Treatment failure (n, %)** ##				
**Overall**	235 (10.4)	390 (9.7)	1.09 [0.92, 1.29]	1.08 [0.91, 1.28]
**Subgroups**				
**55-65**	67 (10.0)	109 (8.5)	1.21 [0.88, 1.66]	1.20 [0.87, 1.66]
**65-75**	77 (10.5)	131 (9.7)	1.10 [0.82, 1.48]	1.09 [0.81, 1.47]
**≥75**	91 (10.6)	150 (10.7)	0.98 [0.75, 1.29]	1.00 [0.76, 1.32]

#Which was defined by new prescription of prednisone or prednisolone within a period of 15 to 31 days after index date. ##Which was defined by a new prescription of antibiotics within a period of 15 to 31 days after index date.
OR = odds ratio; CI = confidence interval;
*Adjusted for age, SABA, LABA/ICS, theophylline.

Table S7. Sensitivity analysis: odds ratio for treatment failure of first exacerbation in COPD outpatients stratified by age groups

	**Doxycycline (n=2261)**	**Reference (n=4039)**	**Crude OR (95% CI)**	**Adjusted OR* (95% CI)**	**P-value**
**Treatment failure (n, %) in study period 2005-2015**					
**Overall**	200 (14.6)	331 (14.0)	1.05 [0.87, 1.27]	1.03 [0.85, 1.25]	0.76
**Subgroups**					
55-64 (1235)	62 (14.8)	90 (11.0)	1.41 [0.99, 1.98]	1.42 [1.00, 2.01]	0.05
65-74 (1160)	64 (15.3)	99 (13.3)	1.18 [0.84, 1.66]	1.18 [0.84, 1.66]	0.35
≥75 (1336)	74 (13.9)	142 (17.7)	0.75 [0.56, 1.02]	0.75 [0.55, 1.01]	0.06
**Treatment failure (n, %) in study period 1994-2004**					
**Overall**	154 (17.2)	309 (18.4)	0.92 [0.75, 1.14]	0.91 [0.74, 1.13]	0.40
**Subgroups**					
55-64 (717)	37 (14.9)	76 (16.2)	0.91 [0.59, 1.39]	0.89 [0.58, 1.37]	0.60
65-74 (930)	52 (16.5)	97 (15.8)	1.05 [0.73, 1.52]	1.04 [0.72, 1.50]	0.84
≥75 (922)	65 (19.8)	136 (22.9)	0.83 [0.59, 1.15]	0.84 [0.60, 1.18]	0.32

OR = odds ratio; CI = confidence interval; *Adjusted for age, SABA, LABA/ICS and theophylline.

Supplementary Figure

a) b)

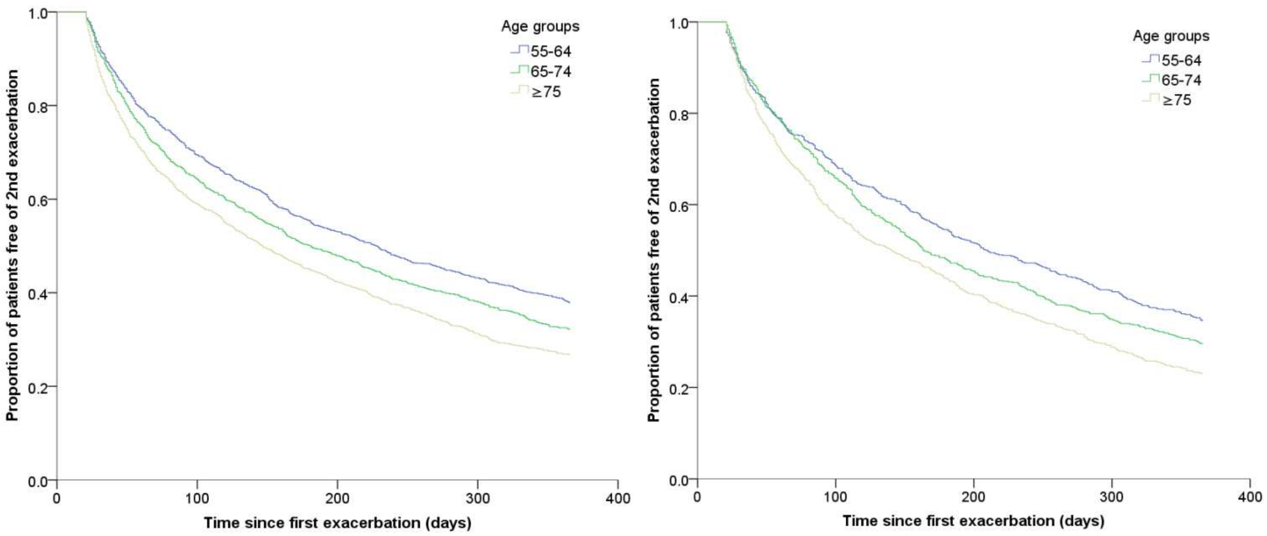

Figure S1. Kaplan-Meier curves showing the proportion of patients free of 2nd exacerbation in COPD outpatients up to 12 months' follow up: a) doxycycline group ($p < 0.01$); b) reference group ($p < 0.01$)

STROBE Statement—Checklist of items that should be included in reports of cohort studies

	Item No	Recommendation	Page No
**Title and abstract**	1	(*a*) Indicate the study's design with a commonly used term in the title or the abstract	1
		(*b*) Provide in the abstract an informative and balanced summary of what was done and what was found	2
**Introduction**			
Background/rationale	2	Explain the scientific background and rationale for the investigation being reported	4
Objectives	3	State specific objectives, including any prespecified hypotheses	5
**Methods**			
Study design	4	Present key elements of study design early in the paper	5
Setting	5	Describe the setting, locations, and relevant dates, including periods of recruitment, exposure, follow-up, and data collection	5, 6
Participants	6	(*a*) Give the eligibility criteria, and the sources and methods of selection of participants. Describe methods of follow-up	5, 6
		(*b*) For matched studies, give matching criteria and number of exposed and unexposed	N/A
Variables	7	Clearly define all outcomes, exposures, predictors, potential confounders, and effect modifiers. Give diagnostic criteria, if applicable	6, 7
Data sources/ measurement	8*	For each variable of interest, give sources of data and details of methods of assessment (measurement). Describe comparability of assessment methods if there is more than one group	5, 6, 7
Bias	9	Describe any efforts to address potential sources of bias	7
Study size	10	Explain how the study size was arrived at	N/A
Quantitative variables	11	Explain how quantitative variables were handled in the analyses. If applicable, describe which groupings were chosen and why	7
Statistical methods	12	(*a*) Describe all statistical methods, including those used to control for confounding	7
		(*b*) Describe any methods used to examine subgroups and interactions	7
		(*c*) Explain how missing data were addressed	N/A
		(*d*) If applicable, explain how loss to follow-up was addressed	N/A
		(*e*) Describe any sensitivity analyses	6
**Results**			
Participants	13*	(a) Report numbers of individuals at each stage of study—eg numbers potentially eligible, examined for eligibility, confirmed eligible, included in the study, completing follow-up, and analysed	8
		(b) Give reasons for non-participation at each stage	8
		(c) Consider use of a flow diagram	22
Descriptive data	14*	(a) Give characteristics of study participants (eg demographic, clinical, social) and information on exposures and potential confounders	8
		(b) Indicate number of participants with missing data for each variable of interest	N/A
		(c) Summarise follow-up time (eg, average and total amount)	8, 9
Outcome data	15*	Report numbers of outcome events or summary measures over time	8, 9
**Discussion**			
Key results	18	Summarise key results with reference to study objectives	10,11

Limitations	19	Discuss limitations of the study, taking into account sources of potential bias or imprecision. Discuss both direction and magnitude of any potential bias	11,12
Interpretation	20	Give a cautious overall interpretation of results considering objectives, limitations, multiplicity of analyses, results from similar studies, and other relevant evidence	10,11
Generalisability	21	Discuss the generalisability (external validity) of the study results	12
**Other information**			
Funding	22	Give the source of funding and the role of the funders for the present study and, if applicable, for the original study on which the present article is based	14

*Give information separately for exposed and unexposed groups.

Note: An Explanation and Elaboration article discusses each checklist item and gives methodological background and published examples of transparent reporting. The STROBE checklist is best used in conjunction with this article (freely available on the Web sites of PLoS Medicine at http://www.plosmedicine.org/, Annals of Internal Medicine at http://www.annals.org/, and Epidemiology at http://www.epidem.com/). Information on the STROBE Initiative is available at http://www.strobe-statement.org.
